# Myocardial perfusion scintigraphy dosimetry: optimal use of SPECT and SPECT/CT technologies in stress-first imaging protocol

**DOI:** 10.1007/s40336-016-0212-9

**Published:** 2016-10-31

**Authors:** M. Lecchi, S. Malaspina, C. Scabbio, V. Gaudieri, A. Del Sole

**Affiliations:** 1 Health Physics, San Paolo Hospital, Via Antonio di Rudini, 20142 Milan, Italy; 2 Department of Health Sciences, University of Milan, Milan, Italy; 3 Nuclear Medicine Unit, Department of Diagnostic Services, ASST Santi Paolo e Carlo, Milan, Italy; 4 Department of Advanced Biomedical Sciences, University Federico II, Naples, Italy

**Keywords:** Myocardial perfusion imaging, SPECT, CT, Dose reduction, Patient dose, Radiation exposure

## Abstract

**Purpose:**

Over the past decade, nuclear medicine experts have been seeking to minimize patient exposure to radiation in myocardial perfusion scintigraphy (MPS). This review describes the latest technological innovations in MPS, particularly with regard to dose reduction.

**Methods:**

We searched in PubMed for original clinical papers in English, published after 2008, using the following research criteria: (dose) and ((reduction) or (reducing)) and ((myocardial) or (cardiac) or (heart)) and ((nuclear medicine) or (nuclear imaging) or (radionuclide) or (scintigraphy) or (SPET) or (SPECT)). Thereafter, recent reviews on the topic were considered and other relevant clinical papers were added to the results.

**Results:**

Of 202 non-duplicate articles, 17 were included. To these, another eight papers cited in recent reviews were added. By optimizing the features of software, i.e., through algorithms for iterative reconstruction with resolution recovery (IRRs), and hardware, i.e., scanners and collimators, and by preferring, unless otherwise indicated, the use of stress-first imaging protocols, it has become possible to reduce the effective dose by at least 50% in stress/rest protocols, and by up to 89% in patients undergoing a diagnostic stress-only study with new technology. With today’s SPECT/CT systems, the use of a stress-first protocol can conveniently be performed, resulting in an overall dose reduction of about 35% if two-thirds of stress-first examinations were considered definitively normal.

**Conclusion:**

Using innovative gamma cameras, collimators and software, as well as, unless otherwise indicated, stress-first imaging protocols, it has become possible to reduce significantly the effective dose in a high percentage of patients, even when X-ray CT scanning is performed for attenuation correction.

## Introduction

Radiological and nuclear medicine procedures are the leading cause of population radiation exposure in Western countries, and there is concern over their potential long-term effects on patient health [1]. Even though, based on the linear no-threshold model, radiation-induced cancer at low doses is probabilistic, it has been demonstrated that this risk increases after exposure to a cumulative dose of radiation greater than 100 mSv [2], a level that can easily be reached through repeated investigations with ionizing radiation.

Among the various procedures involving the use of ionizing radiation, myocardial perfusion scintigraphy (MPS) alone is responsible for over 22% of the total effective dose from all medical imaging procedures in the United States [3]. Therefore, over the past decade, minimization of patient exposure to radiation in nuclear cardiology has become a priority not only for nuclear medicine physicians and health physicists, but also for technologists who perform these scans on a daily basis [4, 5]. This objective can be pursued by adhering to the two main principles of radiation protection: justification of the procedure and optimization of the patient dose in relation to the available technology.

It is to be noted that the prevalence of non-pathological MPS findings may be as high as 35% in patients with known coronary artery disease (CAD) and as high as 81% in those without known CAD [6]. Therefore, conservative strategies promoting radiation protection, such as the use of “stress-first” studies, should, providing there is adequate justification, be preferred for the evaluation of patients, since subsequent rest studies may be avoided in those with a negative stress MPS (stress-only protocol).

The replacement (with some specific exceptions) of ^201^Tl-chloride with two ^99m^Tc-labeled radiopharmaceuticals (physical half-time = 6 h), namely ^99m^Tc-sestamibi and ^99m^Tc-tetrofosmi, has made a major contribution to dose optimization in MPS. More recently, the possibility of carrying out the image acquisition with cardiac dedicated systems rather than with general-purpose (GP) gamma cameras has emerged, and this represents another step forward [7].

Depending on the clinical question and the camera used, MPS studies may require either one acquisition under stress, or two acquisitions (one under stress and one under rest). Accordingly, the imaging protocol may be a one-day stress-only study (one acquisition) or either a two-day imaging protocol or a one-day stress-first protocol (each involving two acquisitions) [8]. Moreover, if a patient has a large body size, this can result in a higher proportion of emitted photons being attenuated within the patient. Therefore, clinical MPS protocols should take into account the patient’s weight or body mass index (BMI) [9].

The optimal amount of activity to be administered to the single patient is determined taking all of the above variables into account. On this basis, the recommended activities, per single scan, according to the American Society of Nuclear Cardiology (ASNC), may range from 148 MBq (stress-only protocol, new technology, BMI = 25 kg/m^2^) to 1332 MBq (second injection in a one-day stress/rest protocol, GP gamma camera, BMI >35 kg/m^2^), resulting in effective doses of between 1.0 mSv and 10.5 mSv [7]. When rest and stress studies are both performed on the same day, the MPS dose may be as low as 4.5 mSv in subjects with BMI = 25 kg/m^2^, providing the recommended procedure is followed and new technologies are employed; conversely, the patient dose can reach 13.5 mSv when using a GP gamma camera in subjects with a BMI >35 kg/m^2^.

Therefore, in this setting, the use of innovative gamma cameras, collimators and software is crucial. By optimizing the features of the software, i.e., through algorithms for iterative reconstruction with resolution recovery (IRRs), and hardware, i.e., the scanners and collimators, and by preferring, unless otherwise indicated, the use of stress-first imaging protocols, it has become possible to reduce the effective dose by at least 50% in stress/rest protocols, and by up to 89% in patients undergoing a diagnostic stress-only study with new technology.

The development of high-efficiency cardiac dedicated scanners equipped with cadmium–zinc–telluride (CZT) detectors has been paralleled by the development of single photon emission computed tomography (SPECT) systems combined with X-ray computed tomography (CT) scanners, mostly employed for attenuation correction (AC) of the emission data. Thus, although the advanced technologies may allow reductions in patient doses, the new combined scanners, due to the CT component, might actually lead to an increase in radiation exposure [10]. The optimized dose from a single CT scan for AC is in the order of 0.3–1.3 mSv [11] and, therefore, it is not negligible, when compared with the dose from the injected radiopharmaceutical, especially for low-dose protocols. Whether or not AC is actually needed is a long-debated and still unresolved question [12], which applies not only to MPS but also other cardiac SPECT examinations, such as evaluation of the sympathetic innervation [13].

The impact of stress-first/stress-only protocols and new technologies, with and without CT, on the patient dose in MPS studies are summarized in Table 1 for normal-weight patients and in Table 2 for obese patients. These doses are based on the very recent ASNC imaging guidelines [7] and on epidemiological data available from a large cohort study [6].

**Table 1:** Mean effective dose among different imaging protocols and SPECT technologies, with and without CT, in normal-weight patients undergoing MPS studies with a normal rate of 66%. Data from [6] and [10]

Patient size	Protocol	Conventional gamma camera	Newer technology	New technology + CT***
BMI = 25 kg/m^2^	For all patients 1-day stress/rest	9.0 mSv (A)	4.5 mSv (B) −50% vs A	5.5 mSv −39% vs A +22% vs B
	1/3 patients 1 day stress/rest plus 2/3 patients stress only*	Not used	2.2** mSv −76% vs A −51% vs B	3.2 mSv −64% vs A −29% vs B

* Stress dose = 1 mSv

** Used with prone-supine or upright-supine imaging

*** CT dose of 1 mSv for stress study (total stress dose = 2 mSv)

**Table 2:** Mean effective dose among different imaging protocols and SPECT technologies, with and without CT, for obese patients undergoing MPS studies with a normal rate of 66%. Data from [6] and [10]

Patient size	Protocol	Conventional gamma camera	Newer technology	New technology + CT***
BMI >35 kg/m^2^	For all patients 1-day stress/rest	13.5 mSv (A)	6.7 mSv (B) −50% vs A	7.7 mSv −43% vs A +15% vs B
	1/3 of patients 1 day stress/rest plus 2/3 of patients stress only*	Not used	3.2** mSv −76% vs A −52% vs B	4.2 mSv −69% vs A −37% vs B

* Stress dose = 1.5 mSv

** Used with prone-supine or upright-supine imaging

*** CT dose of 1 mSv for stress study (total stress dose = 2.5 mSv)

The features of the new technologies in relation to dose optimization are described in the following paragraphs.

### Dose reduction in MPS: IRR algorithms

MPS is commonly performed using GP dual-head gamma cameras based on NaI(Tl) scintillation detectors with photomultiplier tube array and high-resolution parallel-hole collimators. However, limitations of these SPECT systems—namely their low count sensitivity and poor spatial resolution—mean that it is necessary to administer activities in the range of 296–444 MBq for the first radiopharmaceutical injection, and three times as much for the second injection on the same day, and in most cases to perform both rest and stress studies, due to the low image quality and presence of attenuation artifacts when no form of AC is applied (e.g., Gd-153 line source, CT or supine and prone imaging) [14]. But this procedure may entail injected total activities (summing both rest and stress injected activities) of 1.184–1.776 MBq and doses of up to 13.5 mSv per patient, with acquisitions lasting approximately for 15 min, and overall study times of up to 4 h per patient in the case of one-day stress/rest protocols.

**Fig. 1:**
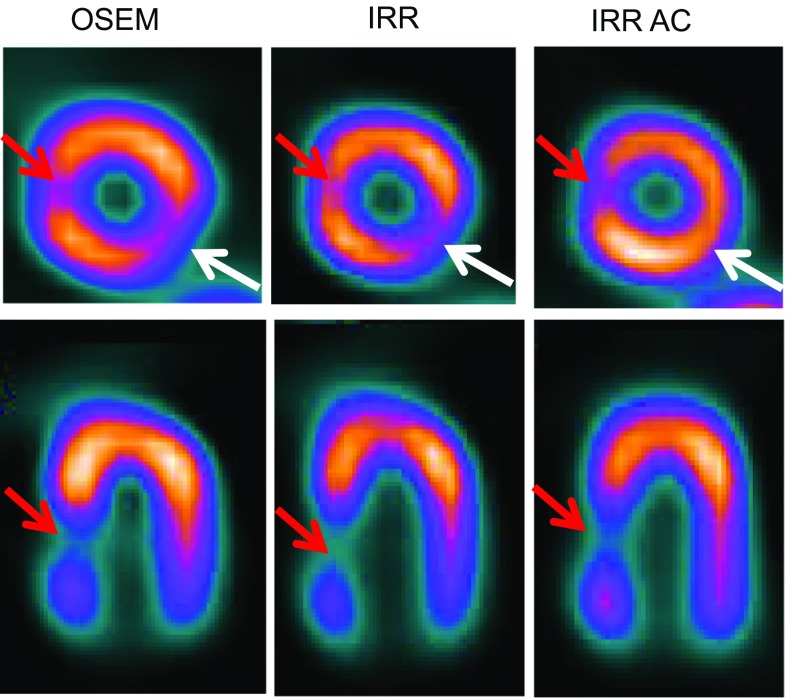
Comparison between different reconstruction protocols for left ventricular short-axis (*top*) and horizontal long-axis (*bottom*) images of an anthropomorphic phantom scan with in vivo reference count statistics (Torso Phantom™ and Cardiac Insert™, Data Spectrum Corporation). The *white arrows* show an attenuation artifact in the uncorrected images. The *red arrows* show a true perfusion defect in the phantom. *OSEM* ordered-subset expectation maximization, *IRR* iterative reconstruction with resolution recovery, *NC* no correction, *AC* attenuation correction

**Fig. 2:**
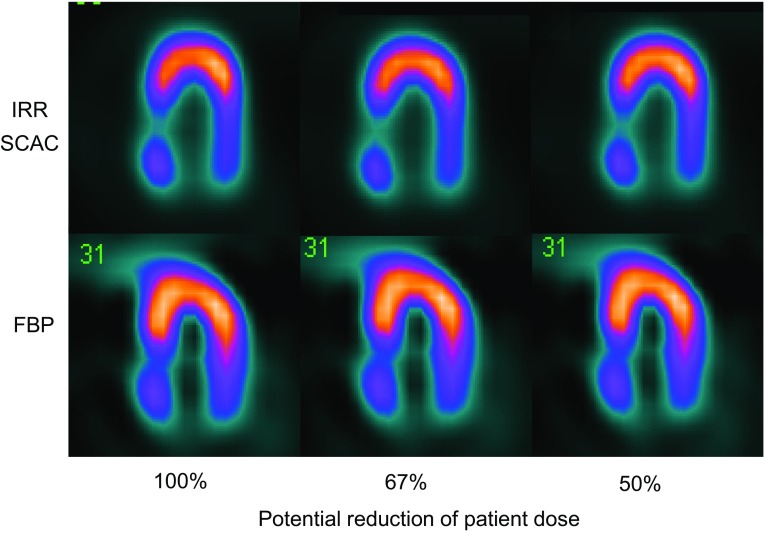
Left ventricular horizontal long-axis images of an anthropomorphic phantom (Torso Phantom™ and Cardiac Insert™, Data Spectrum Corporation) acquired at different potential percentages of dose reduction. Images are reconstructed with iterative reconstruction algorithm with resolution recovery plus scatter and attenuation corrections (*top*) and with filtered back projection reconstruction (*bottom*)

The IRR algorithms include resolution recovery and noise compensation in the iterative reconstruction (IR) process, and they make it possible to reduce the study count statistics, without degradation of the image quality, to 25% of the reference value of traditional IR algorithms, such as ordered-subset expectation maximization (OSEM) [15]. The use of CT-based AC results in a further reduction of background noise and in an increase of uniformity also in the polar-map representation of the left ventricular signal [16] (Figs. 1, 2).

The IRR algorithms were introduced into clinical practice primarily to reduce acquisition times, since fast imaging provides immediate benefits in terms of patient throughput and patient comfort. However, equivalent dose reduction is also possible by performing standard time acquisitions with lower injected activities.

Using the IRR algorithm Astonish™ (Philips), the possibility of decreasing the dose to 25% of the reference value was evaluated in the context of a two-day imaging protocol, without AC (3 mSv for a full examination). The authors found that decreasing radiopharmaceutical activity to 25% of the reference value seemed practicable for normal-weight patients, while an activity reduction limited to 50% was suggested to be more appropriate for overweight and obese patients [17]. Other studies investigated the possibility of halving the dose to patients using the reduced acquisition time to simulate an equivalent reduction of the injected activity [18–20].

Bateman et al., using the Astonish™, performed stress-only imaging with Gd-153 for AC and half-counts, and found this method to provide clinical results equivalent to those of conventional stress/rest scanning without AC [21]. Marcassa et al. [22] used wide-beam reconstruction (WBR) to compare patient effective dose in different dose administration protocols. They found that the additional use of an IRR algorithm made it possible to reduce patient effective dose by 40% (from 9.5 to 5.7 mSv, *p* < 0.001).

With full-time acquisitions (15 min), a 50% dose reduction seems to be the limit for IRR algorithms. With today’s SPECT/CT systems the use of a stress-first protocol can conveniently be performed. If two-thirds of patients were considered definitively normal after the stress study, there would be no need to perform the rest study in these patients. In such a situation, patient dose would decrease by 76%, but the CT dose has to be added, resulting in an overall dose reduction of about 35% (see Tables 1, 2).

### MPS dose reduction: multifocal collimators

An alternative approach, such as the IQ⋅SPECT solution (Siemens), is to adapt a GP gamma camera to the particular application of cardiac imaging using dedicated multifocal collimators (SMARTZOOM), cardio-centric acquisition, and an IRR algorithm optimized for myocardial studies. Compared with conventional parallel collimation, IQ⋅SPECT shows similar global image quality, including resolution, but a fourfold higher sensitivity, which allows the use of either a low-dose or a short-time imaging protocol, or a combination of the two [23].

However, attenuation artifacts are more marked than with parallel collimation, as well as being unpredictable and position dependent; for this reason, AC is mandatory for the IQ⋅SPECT users [24].

Very recently, Lyon et al., using IQ⋅SPECT technology, found that one-day rest–stress gated SPECT/CT, quantitative stress MPS imaging is possible with 50% of the standard injected activity in 50% of the time with respect to imaging using conventional gamma cameras [25].

To the best of our knowledge, no studies have been performed with IQ⋅SPECT configuration to evaluate the feasibility of stress-first/stress-only imaging protocol.

### MPS dose reduction: CZT detectors

Technological advances have led to the development of solid-state detectors, in particular CZT tomographs equipped with detector geometry optimized for cardiac imaging, which allow a greater count sensitivity (up to 7 times) with improved spatial resolution (over 2 times) compared with GP cameras [26]. Thanks to this sensitivity, CZT cameras provide several advantages and opportunities over GP cameras. First, they make it possible to reduce either the amount of injected activity or the acquisition time, or both, and thus to optimize both. Second, due to the improved image quality of the stress study, i.e., their fewer artifacts, stress studies have a higher prognostic value and allow the rest study to be avoided in the presence of conclusive normal stress scans.

Two systems (Discovery NM 530c, General Electric and D-SPECT, Spectrum Dynamics) having the same configuration as multiple CZT detectors, but coupled with different high-sensitivity collimators (multipinhole vs high-sensitivity parallel-hole collimators, respectively) and IRR algorithms, have been introduced on the market [27, 28]. A comparative study, performed with an anthropomorphic phantom mimicking the thorax of a normal adult, has shown relatively similar physical performance of the two CZT cameras, even though the corresponding images of the left ventricle insert were significantly different, revealing a smaller wall thickness with the D-SPECT than with the Discovery NM 530c (13.6 ± 0.7 vs 19.6 ± 1.3 mm, respectively) [29]. It is unlikely, however, that such differences have a significant impact on diagnostic accuracy.

The study by Duvall et al. was among the first to demonstrate that a low-dose one-day ^99m^Tc-sestamibi protocol (185 MBq for rest and 555 MBq for stress) with 5-min acquisition time is feasible with the Discovery NM530c, without compromising image quality and diagnostic accuracy. They showed that effective doses to patients could be reduced by 50% compared to those associated with a conventional gamma camera [30].

Other studies have confirmed the performance of CZT systems in halving the patient dose and, at the same time, in reducing the acquisition time to less than 10 min (low-dose ultrafast protocol) [31–33].

**Fig. 3:**
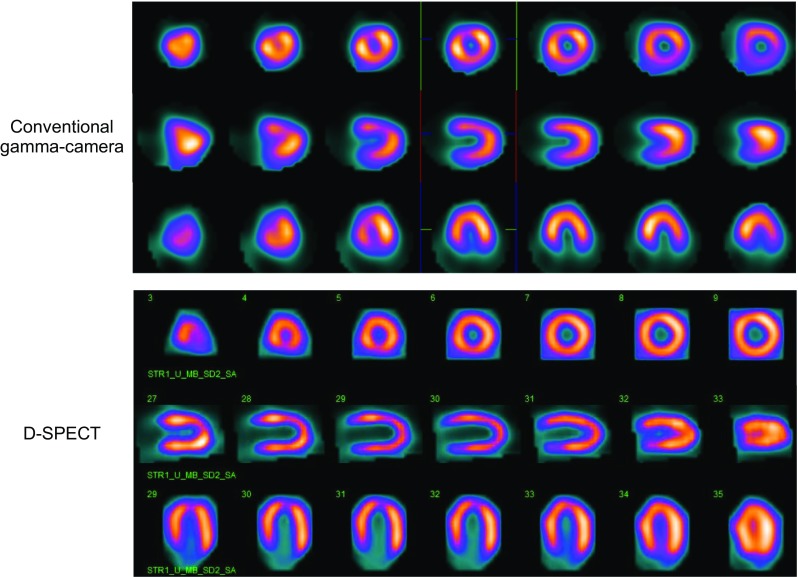
Comparison between stress MPS images of a 48-year-old woman with hypertension, dyslipidemia and familiarity for CAD acquired with two different gamma cameras. The images acquired with a conventional dual-head GP gamma camera reveal a defect in anterior wall due to attenuation by left breast (*top*), while the stress images with a dedicated CZT gamma camera (D-SPECT) and upright patient position show homogenous left ventricular perfusion (*bottom*)

**Fig. 4:**
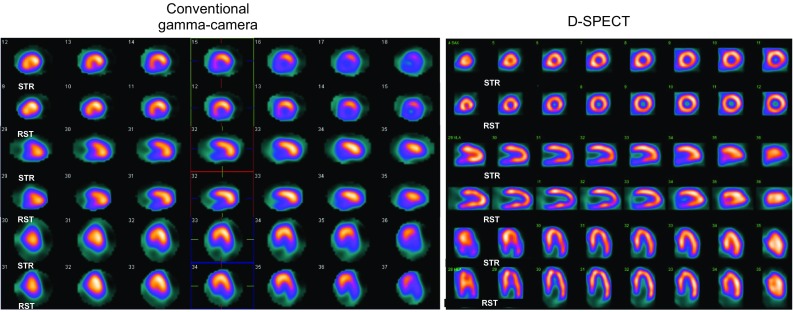
Comparison between stress and rest MPS images of an obese 56-year-old man (BMI 35.5 kg/m^2^) with suspected CAD. The stress images acquired with a conventional dual-head GP gamma camera show a fixed inferior wall perfusion defect due to diaphragmatic attenuation (*left*), while the stress/rest images with a dedicated CZT gamma camera (D-SPECT) and upright patient position show normal perfusion (*right*)

The possibility of using different patient positions, such as prone or upright, alone or in addition to the supine position, to reduce attenuation artifacts, due to the individual patient’s body conformation and weight, was successfully investigated in stress-first procedures and one-day protocols with CZT systems (Figs. 3, 4) [34, 35].

Einstein et al. demonstrated that a stress-only imaging protocol using an advanced CZT system and a fixed activity could be performed in more than two-thirds of patients with an effective dose averaging 1 mSv, in a study time that, on average, amounted to less than 2 h to complete the full MPS examination, and that it had excellent prognostic value [36]. One-day stress-first MPS with 50% radiation reduction and a very low stress dose (<2 mSv) using CZT technology and quantitative supine and prone analysis provided a high diagnostic value, similar to standard dose MPS [37].

However, few studies adapted the injected activities to patient weight or BMI. In the study by Hindorf et al., patients received an intravenous injection of 2.5 MBq kg^−1^ body weight of ^99m^Tc-tetrofosmin for the stress examination. The rest examination was performed only when the stress images were interpreted as abnormal with an injection of 4 MBq kg^−1^ [33]. Very recently, Oddstig et al. demonstrated that the linear low-dose weight-adjusted protocol of 2.5 MBq/kg can be applied over a wide range of body weights (51–193 kg, BMI 18–58) without loss of counts or image quality, and resulting in a significant reduction in radiation exposure to obese patients. In the case of a patient weighing 120 kg, the effective dose for the stress examination would decrease to 2.1 mSv (300 MBq) [38].

The CZT systems are available with up to 64-slice CT configuration (GE NM 570c). Very recently, Palyo et al. demonstrated that an ultra-low dose (<190 MBq) in the stress examination, even with short imaging times (<6 min), is feasible using a hybrid CZT-SPECT/CT camera without compromising image quality or significantly altering quantification of myocardial perfusion or left ventricular function [39].

However, many institutions may not have access to a CZT-SPECT system. Although these novel scanners offer high-quality imaging with a low radiation dose, they remain expensive, and are used in the clinical setting only for cardiac imaging [40].

## Conclusion

Using innovative gamma cameras, collimators and software, as well as, unless otherwise indicated, stress-first imaging protocols, it has become possible to reduce significantly the effective dose in a high percentage of patients.

By combining new SPECT technologies with X-ray-CT-based AC, MPS stress-only studies can conveniently be performed and likely reduce the incidence of attenuation artifacts. Although single patients may actually receive an increased effective dose due to X-ray CT scanning, in the order of 1 mSv when the CT scan is optimized for AC, application of the stress-first protocol (SPECT + CT) allows the reduction of the dose over the entire population of patients undergoing MPS evaluation, since a conclusive normal stress study will exclude coronary artery disease and eliminate the need to perform a rest examination. On the other hand, an abnormal MPS will still require a rest evaluation to differentiate ischemia from scar. To further reduce the dose to the patients, the rest examination could be acquired without AC. In this case, the rest images should be compared with the corresponding non-corrected stress images.

## References

[1] Picano E, Vañó E, Rehani MM, Cuocolo A, Mont L, Bodi V (2014). The appropriate and justified use of medical radiation in cardiovascular imaging: a position document of the ESC Associations of Cardiovascular Imaging, Percutaneous Cardiovascular Interventions and Electrophysiology. Eur Heart J.

[2] Le Guludec D, Aigueperse J (2016). Dose optimization: a major challenge for acceptability of nuclear medicine. Clin Transl Imaging..

[3] Fazel R, Krumholz HM, Wang Y, Ross JS, Chen J, Ting HH (2009). Exposure to low-dose ionizing radiation from medical imaging procedures. N Engl J Med.

[4] Cerqueira MD, Allman KC, Ficaro EP, Hansen CL, Nichols KJ, Thompson RC (2010). Recommendations for reducing radiation exposure in myocardial perfusion imaging. J Nucl Cardiol..

[5] Slomka PJ, Dey D, Duvall WL, Henzlova MJ, Berman DS, Germano G (2012). Advances in nuclear cardiac instrumentation with a view towards reduced radiation exposure. Curr Cardiol Rep.

[6] Duvall WL, Rai M, Ahlberg AW, O’Sullivan DM, Henzlova MJ (2015). A multi-center assessment of the temporal trends in myocardial perfusion imaging. J Nucl Cardiol..

[7] Henzlova MJ, Duvall WL, Einstein AJ, Travin MI, Verberne HJ (2016). ASNC imaging guidelines for SPECT nuclear cardiology procedures: Stress, protocols, and tracers. J Nucl Cardiol..

[8] DePuey EG, Mahmarian JJ, Miller TD, Einstein AJ, Hansen CL, Holly TA (2012). Patient-centered imaging. J Nucl Cardiol..

[9] Cuocolo A (2016). Challenges and opportunities of noninvasive cardiac imaging in obesity. J Nucl Cardiol.

[10] Ferrari M, De Marco P, Origgi D, Pedroli G (2014). SPECT/CT radiation dosimetry. Clin Transl Imaging..

[11] Einstein AJ, Johnson LL, Bokhari S, Son J, Thompson RC, Bateman TM (2010). Agreement of visual estimation of coronary artery calcium from low-dose CT attenuation correction scans in hybrid PET/CT and SPECT/CT with standard Agatston score. J Am Coll Cardiol.

[12] Cuocolo A (2011). Attenuation correction for myocardial perfusion SPECT imaging: still a controversial issue. Eur J Nucl Med Mol Imaging.

[13] Pellegrino T, Piscopo V, Boemio A, Russo B, De Matteis G, Pellegrino S (2015). Impact of obesity and acquisition protocol on (123)I-metaiodobenzylguanidine indexes of cardiac sympathetic innervation. Quant Imaging Med Surg..

[14] Hussain N, Parker MW, Henzlova MJ, Duvall WL (2016). Stress-first Myocardial Perfusion Imaging. Cardiol Clin.

[15] Zoccarato O, Scabbio C, De Ponti E, Matheoud R, Leva L, Morzenti S (2014). Comparative analysis of iterative reconstruction algorithms with resolution recovery for cardiac SPECT studies. A multi-center phantom study. J Nucl Cardiol..

[16] Zoccarato O, Marcassa C, Lizio D, Leva L, Lucignani G, Savi A (2016). Differences in polar-map patterns using the novel technologies for myocardial perfusion imaging. J Nucl Cardiol.

[17] Lecchi M, Martinelli I, Zoccarato O, Maioli C, Lucignani G, Del Sole A (2016). Comparative analysis of full-time, half-time, and quarter-time myocardial ECG-gated SPECT quantification in normal-weight and overweight patients. J Nucl Cardiol.

[18] Valenta I, Treyer V, Husmann L, Gaemperli O, Schindler MJ, Herzog BA (2010). New reconstruction algorithm allows shortened acquisition time for myocardial perfusion SPECT. Eur J Nucl Med Mol Imaging.

[19] DePuey EG, Bommireddipalli S, Clark J, Leykekhman A, Thompson LB, Friedman M (2011). A comparison of the image quality of full-time myocardial perfusion SPECT vs wide beam reconstruction half-time and half-dose SPECT. J Nucl Cardiol..

[20] Marcassa C, Campini R, Zoccarato O, Calza P (2011). Wide beam reconstruction for half-dose or half-time cardiac gated SPECT acquisitions: optimization of resources and reduction in radiation exposure. Eur J Nucl Med Mol Imaging.

[21] Bateman TM, Heller GV, McGhie AI, Courter SA, Golub RA, Case JA (2009). Multicenter investigation comparing a highly efficient half-time stress-only attenuation correction approach against standard rest-stress Tc-99 m SPECT imaging. J Nucl Cardiol..

[22] Marcassa C, Zoccarato O, Calza P, Campini R (2013). Temporal evolution of administered activity in cardiac gated SPECT and patients’ effective dose: analysis of an historical series. Eur J Nucl Med Mol Imaging.

[23] Caobelli F, Kaiser SR, Thackeray JT, Bengel FM, Chieregato M, Soffientini A (2014). IQ SPECT allows a significant reduction in administered dose and acquisition time for myocardial perfusion imaging: evidence from a phantom study. J Nucl Med.

[24] Gremillet E, Agostini D (2016). How to use cardiac IQ•SPECT routinely? An overview of tips and tricks from practical experience to the literature. Eur J Nucl Med Mol Imaging.

[25] Lyon MC, Foster C, Ding X, Dorbala S, Spence D, Bhattacharya M (2016). Dose reduction in half-time myocardial perfusion SPECT-CT with multifocal collimation. J Nucl Cardiol..

[26] Imbert L, Perrin M, Verger A, Marie P-Y (2016). Dose optimization for myocardial perfusion CZT-SPECT imaging: why and how?. Clin Transl Imaging..

[27] Erlandsson K, Kacperski K, van Gramberg D, Hutton BF (2009). Performance evaluation of D-SPECT: a novel SPECT system for nuclear cardiology. Phys Med Biol.

[28] Bocher M, Blevis IM, Tsukerman L, Shrem Y, Kovalski G, Volokh L (2010). A fast cardiac gamma camera with dynamic SPECT capabilities: design, system validation and future potential. Eur J Nucl Med Mol Imaging.

[29] Zoccarato O, Lizio D, Savi A, Indovina L, Scabbio C, Leva L (2016). Comparative analysis of cadmium-zincum-telluride cameras dedicated to myocardial perfusion SPECT: a phantom study. J Nucl Cardiol..

[30] Duvall WL, Croft LB, Godiwala T, Ginsberg E, George T, Henzlova MJ (2010). Reduced isotope dose with rapid SPECT MPI imaging: initial experience with a CZT SPECT camera. J Nucl Cardiol.

[31] Gunalp B (2015). Role of cardiac ultrafast cameras with CZT solid-state detectors and software developments on radiation absorbed dose reduction to the patients. Radiat Prot Dosimetry.

[32] Gimelli A, Bottai M, Quaranta A, Giorgetti A, Genovesi D, Marzullo P (2013). Gender differences in the evaluation of coronary artery disease with a cadmium-zinc telluride camera. Eur J Nucl Med Mol Imaging.

[33] Hindorf C, Oddstig J, Hedeer F, Hansson MJ, Jögi J, Engblom H (2014). Importance of correct patient positioning in myocardial perfusion SPECT when using a CZT camera. J Nucl Cardiol.

[34] Perrin M, Djaballah W, Moulin F, Claudin M, Veran N, Imbert L, et al (2015) Stress-first protocol for myocardial perfusion SPECT imaging with semiconductor cameras: high diagnostic performances with significant reduction in patient radiation doses. Eur J Nucl Med Mol Imaging 1004–1011 10.1007/s00259-015-3016-7 25711177

[35] Chaudhry W, Hussain N, Ahlberg AW, Croft LB, Fernandez AB, Parker MW (2015). Multicenter evaluation of stress-first myocardial perfusion image triage by nuclear technologists and automated quantification. J Nucl Cardiol.

[36] Einstein AJ, Johnson LL, DeLuca AJ, Kontak AC, Groves DW, Stant J (2015). Radiation dose and prognosis of ultra-low-dose stress-first myocardial perfusion SPECT in patients with chest pain using a high-efficiency camera. J Nucl Med.

[37] Sharir T, Pinskiy M, Pardes A, Rochman A, Prokhorov V, Kovalski G (2016). Comparison of the diagnostic accuracies of very low stress-dose with standard-dose myocardial perfusion imaging: automated quantification of one-day, stress-first SPECT using a CZT camera. J Nucl Cardiol.

[38] Oddstig J, Hindorf C, Hedeer F, Jögi J, Arheden H, Hansson MJ (2016). The radiation dose to overweighted patients undergoing myocardial perfusion SPECT can be significantly reduced: validation of a linear weight-adjusted activity administration protocol. J Nucl Cardiol.

[39] Palyo RJ, Sinusas AJ, Liu Y-H (2016). High-sensitivity and high-resolution SPECT/CT systems provide substantial dose reduction without compromising quantitative precision for assessment of myocardial perfusion and function. J Nucl Med.

[40] DePuey EG (2016). Traditional gamma cameras are preferred. J Nucl Cardiol..

